# Prognostic analysis and risk stratification of lung adenocarcinoma undergoing EGFR-TKI therapy with time-serial CT-based radiomics signature

**DOI:** 10.1007/s00330-022-09123-5

**Published:** 2022-09-27

**Authors:** Xiaobo Zhang, Bingfeng Lu, Xinguan Yang, Dong Lan, Shushen Lin, Zhipeng Zhou, Kai Li, Dong Deng, Peng Peng, Zisan Zeng, Liling Long

**Affiliations:** 1 Department of Radiology, The First Affiliated Hospital of Guangxi Medical University, No.6 Shuangyong Road, Qingxiu District, Nanning, 530021 Guangxi China; 2 Department of Radiology, The Second Affiliated Hospital of Guangxi Medical University, Nanning, Guangxi China; 3 Department of Radiology, Guilin People’s Hospital, Guilin, Guangxi China; 4 Department of Oncology, The First Affiliated Hospital of Guangxi Medical University, Nanning, Guangxi China; 5 Siemens Healthineers, Shanghai, China; 6 Department of Radiology, Affiliated Hospital of Guilin Medical University, Guilin, Guangxi China

**Keywords:** Radiomics, Lung adenocarcinoma, EGFR-TKI, Time-serial computed tomography, Peritumoral feature

## Abstract

**Objectives:**

To evaluate the value of time-serial CT radiomics features in predicting progression-free survival (PFS) for lung adenocarcinoma (LUAD) patients after epidermal growth factor receptor-tyrosine kinase inhibitors (EGFR-TKIs) therapy.

**Materials and methods:**

LUAD patients treated with EGFR-TKIs were retrospectively included from three independent institutes and divided into training and validation cohorts. Intratumoral and peritumoral features were extracted from time-serial non-contrast chest CT (including pre-therapy and first follow-up images); moreover, the percentage variation per unit time (day) was introduced to adjust for the different follow-up periods of each patient. Test-retest was performed to exclude irreproducible features, while the Boruta algorithm was used to select critical radiomics features. Radiomics signatures were constructed with random forest survival models in the training cohort and compared against baseline clinical characteristics through Cox regression and nonparametric testing of concordance indices (C-indices).

**Results:**

The training cohort included 131 patients (74 women, 56.5%) from one institute and the validation cohort encompassed 41 patients (24 women, 58.5%) from two other institutes. The optimal signature contained 10 features and 7 were unit time feature variations. The comprehensive radiomics model outperformed the pre-therapy clinical characteristics in predicting PFS (training: 0.78, 95% CI: [0.72, 0.84] versus 0.55, 95% CI: [0.49, 0.62], *p* < 0.001; validation: 0.72, 95% CI: [0.60, 0.84] versus 0.54, 95% CI: [0.42, 0.66], *p* < 0.001).

**Conclusion:**

Radiomics signature derived from time-serial CT images demonstrated optimal prognostic performance of disease progression. This dynamic imaging biomarker holds the promise of monitoring treatment response and achieving personalized management.

**Key Points:**

• *The intrinsic tumor heterogeneity can be highly dynamic under the therapeutic effect of EGFR-TKI treatment, and the inevitable development of drug resistance may disrupt the duration of clinical benefit. Decision-making remained challenging in practice to detect the emergence of acquired resistance during the early response phase.*

• *Time-serial CT-based radiomics signature integrating intra- and peritumoral features offered the potential to predict progression-free survival for LUAD patients treated with EGFR-TKIs.*

• *The dynamic imaging signature allowed for prognostic risk stratification.*

**Supplementary Information:**

The online version contains supplementary material available at 10.1007/s00330-022-09123-5.

## Introduction

Epidermal growth factor receptor (EGFR) is one of the most targeted driver genes, and its activating mutation is especially prevalent in the Eastern Asian lung adenocarcinoma (LUAD) population [1]. Many studies demonstrated that LUAD patients harboring EGFR mutations could benefit from EGFR tyrosine kinase inhibitor (TKI) therapy, which compared to chemotherapy alone would prolong progression-free survival (PFS) [2–6]. However, most of these patients eventually develop progression due to the acquired resistance to EGFR TKIs within 1 year after the initiation of targeted treatment [3–6]. Therefore, accurately predicting disease progression and early identifying patients whose drug resistance developed rapidly are crucial to optimize follow-up strategy and guide subsequent treatment.

Re-biopsy at the targeted lesion is the main method to determine secondary resistance [7, 8]. However, the application of re-biopsy is limited in clinical practice for many reasons including its invasiveness, limited tissue availability [9, 10] and ambiguous timing of such examination [9, 11]. As the result, early prediction and detection of acquired resistance remain challenging.

Radiomics emerged as a popular image analysis method in the past decade, enabling high-throughput feature extraction to quantitatively describe tumor heterogeneity [12–14]. Previous lung cancer studies applied radiomics to differential diagnosis [15, 16], treatment response [17–19], prognostic prediction [20–26], estimation of tumor phenotype [19] and mutation status [27–30]. Most radiomics-based studies have predominantly investigated the association between clinical outcomes and intra-tumoral radiomics features at a single timepoint. For example, Song et al indicated radiomics features extracted from pretherapy CT images had a considerable value on prediction of PFS for stage IV EGFR-mutant non-small cell lung cancer (NSCLC) patients who received EGFR-TKI therapy [20]. However, few studies focused on the prognostic value of radiomics features at multiple timepoints throughout EGFR-TKI therapy for LUAD patients with EGFR mutation [19, 21, 24, 31] or considered the prognostic impact of peritumoral tissue [26]. Fave et al concluded that delta-radiomics features could provide better patient-specific outcome predictions compared with pretreatment features [24]. Meanwhile, Dou et al identified peritumoral radiomics features as significant indicators of distant metastasis in locally advanced NSCLC [26]. Therefore, our study aimed to explore whether a radiomics signature encompassing intra- and peri-tumoral radiomics features from time-serial CT can accurately predict PFS and early stratify the risk of acquired resistance for lung adenocarcinoma patients undergoing EGFR-TKI treatment.

## Materials and methods

### Patient selection

**Fig. 1:**
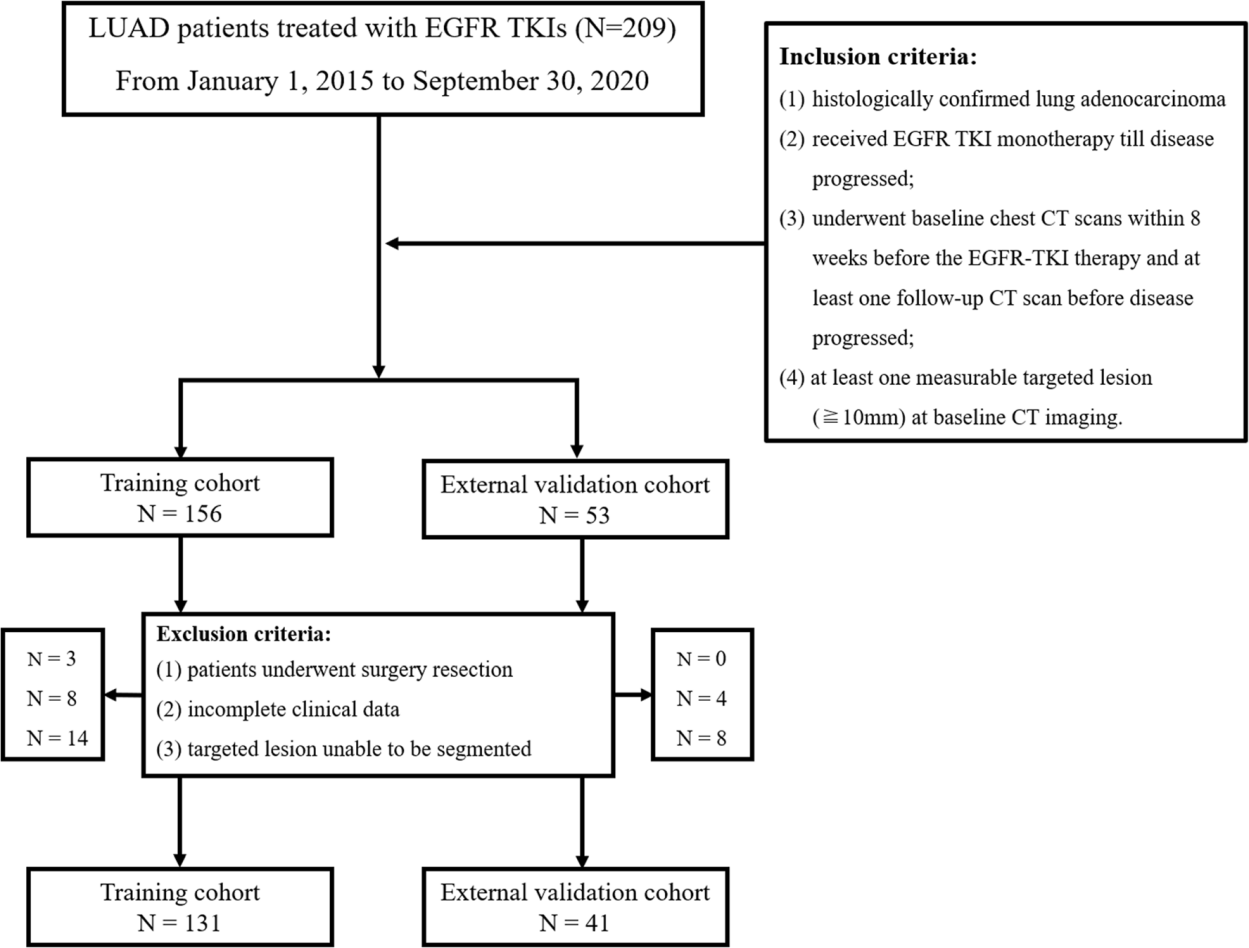
Flow chart of patient selection with inclusion and exclusion criteria

Two hundred and nine lung adenocarcinoma patients who were treated with EGFR TKIs according to the criteria established by the National Comprehensive Cancer Network (NCCN) at three independent institutes between January 1, 2015 and September 30, 2020 were evaluated. The clinical characteristics, such as sex, age, ethnicity, smoking status, administered targeted drug, and TNM stage, were recorded from Hospital Information System (HIS) for all eligible patients. Patients’ serial CT data were obtained from Picture Archiving and Communication Systems (PACS). Inclusion criteria were as follows: (1) histologically confirmed lung adenocarcinoma; (2) received EGFR TKI monotherapy till disease progressed; (3) underwent baseline chest CT scans within 8 weeks before the EGFR-TKI therapy and at least one follow-up CT scan before disease progressed; (4) at least one measurable targeted lesion (≧ 10 mm) at baseline CT imaging. Exclusion criteria were as follows: (1) Patients with history of surgery resection; (2) incomplete clinical data; (3) targeted lesions unable to be segmented (e.g. lesions adjacent to atelectasis or diffuse lesions). A flow chart of patient selection is illustrated in Fig. 1.

### CT examinations, follow-up strategy, and disease progression

All those who were eligible underwent baseline chest CT scans within 8 weeks before the EGFR-TKI therapy. The follow-up interval was every 6–12 weeks according to NCCN guidelines, wherein the first follow-up for response assessment was performed 6 weeks after initiation of therapy. A portion of patients (30/172, 17.4%) didn’t undergo imaging scans at that timepoint. Therefore, the first follow-up CT scan images before disease progression occurred were analyzed.

The response to EGFR-TKI treatment was assessed according to Hayes’ criteria [32] and RECIST version 1.1 [33]. Disease progression was determined as the volume of targeted lesion increased by 20% or the appearance of new lesions. The primary endpoint of this study is disease progression, and PFS was defined as the duration from initiation of EGFR-TKI therapy to disease progression. Death, loss to follow-up, and non-occurrence of outcome were properly censored.

The chest CT examinations were performed with multi-vendor multi-systems at the three institutes. Details regarding the acquisition and reconstruction parameters are presented in Table S1.

### Radiomics feature extraction

**Fig. 2:**
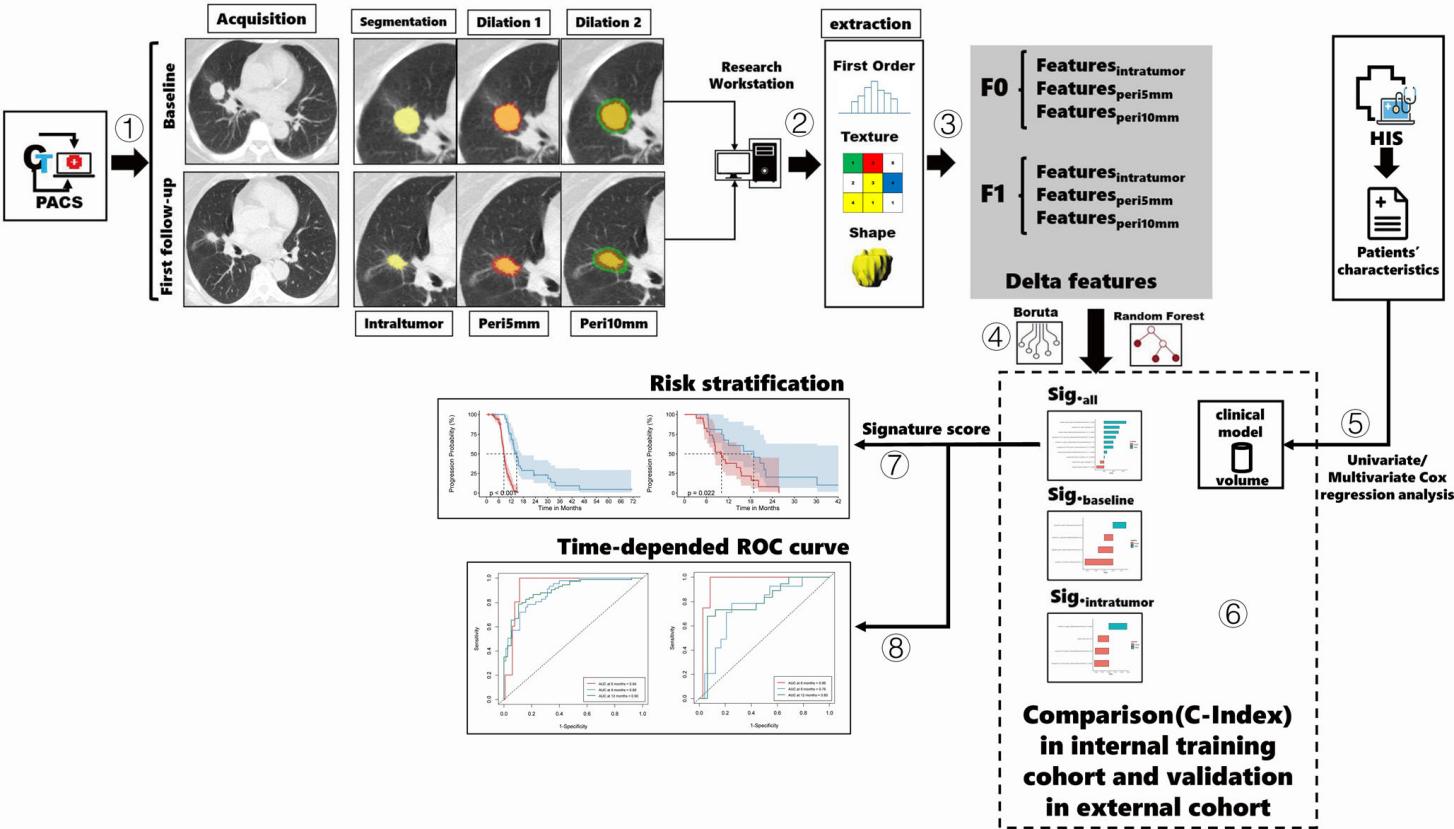
Study workflow. Step 1: Serial CT images including baseline and first follow-up were acquired and segmented. Primary tumor and its peritumoral rings were semi-automatically delineated and manually confirmed. Step 2: Radiomics features based on first-order, shape and texture characteristics were extracted from each segmented region through a prototypical software. Step 3: Test-retest was performed to select stable features with ICC > 0.8 and subsequently compute delta features. Step 4: Feature selection from internal training cohort was conducted with Boruta algorithm to formulate optimal radiomics signatures through random forest survival models. Step 5: Univariate and multivariate Cox regression analysis was performed with all clinical factors and a clinical model was established. Step 6: The progression-predictive performance between models was compared in training cohort and validated in external cohort. Step 7: Risk stratification with the best-performing radiomics signature. Step 8: Time-depended ROC curve was used to evaluate the accuracy of prediction at different time points

Three-dimensional (3D) volumes of interest (VOIs) of primary lesion at baseline (F0) and first follow-up (F1) were segmented semi-automatically by a radiologist with 21 years of experience in chest CT interpretation in a research workstation (Syngo.via Frontier, Radiomics, version 1.2.5, Siemens Healthineers). The radiologist delineated the primary pulmonary lesions in three-dimensional space, and then defined two peritumoral rings (peri_5mm_ and peri_10mm_) based on segmented tumor contour by dilating the lesion borders by 5 mm and 10 mm, respectively (Fig. 2). After border dilation, the radiologist paid close attention to avoid the peritumoral regions from breaching into the chest wall or lung mediastinum and manually modified the segmentations if necessary. Baseline tumor volumes were also recorded from segmentation. The peri_5mm_ ring was obtained by subtracting the region of the primary tumor from the region of dilation_5mm_. In the same way, the peri_10mm_ ring was generated by subtraction between the region of dilation_5mm_ and dilation_10mm_. For each segmented region, the prototypical software outputted 1691 radiomics features based on intensity, shape, and texture characteristics (Table S2). To ensure the reliability and reproducibility of the extracted features, the same radiologist performed test-retest analysis by conducting segmentations on 30 randomly chosen patients a month apart from the initial evaluation. The interclass correlation coefficient (ICC) greater than 0.80 was considered as excellent reliability and subsequently used to exclude features with low intra-observer agreement. Finally, the remaining features were compared between baseline and first follow-up to compute the percentage variation per unit time (Eq. 1, where *Diff*_*time*_ refers to the time difference between two CT scans).

Equation 1 Percentage variation per unit time (day)

1 $$ {Delta}_{radiomics}=\frac{1}{Diff_{time}}\cdotp \frac{Radiomics_{F1}-{Radiomics}_{F0}}{Radiomics_{F0}} $$

### Feature selection and radiomics signature construction

The stable features from baseline, first follow-up, and between-scan variation from the internal training cohort underwent feature selection process with the Boruta algorithm. This algorithm iteratively wrapped around random forest survival analyses to explore all possible subsets of the attributes and finally selects the most important features related to PFS by comparing the importance of the original attributes with the importance achievable at all permutations [34, 35]. Based on the sample size of the training cohort, the ten most important features were refined to build the optimal random forest survival model and obtain the radiomics signature (model_all_) of each patient. Meanwhile, the same feature selection and modeling processes were repeated with baseline imaging features alone (model_baseline_) and intratumoral features alone (model_intratumor_) to understand the added value of the time-serial radiomics features and peritumoral features.

### Risk stratification and prognostic accuracy

LUAD patients were stratified into rapid or slow progression subgroups using the cutoff radiomics signature score determined in the training cohort by maximally selected Log-Rank statistics. The Kaplan-Meier analysis was performed in both training and external validation cohorts to identify the clinical efficacy of our proposed radiomics signature. The prognostic accuracy of the signature at different timepoints was evaluated by the time-dependent receiver operating characteristic (ROC) curve. ROC curves at the 6th, 9th, and 12th months were plotted for both cohorts respectively and the corresponding areas under the curve (AUC) were calculated.

### Statistical analysis

Baseline demographic and clinical characteristics were expressed in means ± standard deviations, medians with interquartile ranges (IQR), and frequencies (percentages) based on normality and continuous nature of the variable. Chi-square test and Wilcoxon’s two-sample test were deployed to compare the patient characteristics between the internal training cohort and the external validation cohort. Cox regression models were built for each radiomics signature to evaluate their PFS-predicting performance. Meanwhile, the clinical parameters underwent a univariate Cox regression analysis and a forward selecting multivariate Cox regression analysis, and the clinical model (model_clinical_) was constructed with the selected variables. Akaike information criterion (AIC) was adopted to screen the optimal clinical model. The model containing the fewest parameters with the lowest AIC value is the most optimal model. Additionally, the best-performing radiomics signature was combined with the clinical model to achieve a clinico-radiomics prediction (model_combined_). Harrell's concordance index (C-Index) in both training and validation cohorts were calculated and compared via a pairwise nonparametric test among radiomics and clinical and combined models. All statistical analyses were conducted using R software (version 3.6.0, http://www.Rproject.org).

## Results

### Patient characteristics

A total of 172 eligible patients were enrolled in this study according to our criteria. A hundred and thirty-one patients from the institute A were designated as the training cohort (74 females, 56.5%). The remanent 41 patients from the other two independent hospitals (24 from institute B and 17 from institute C, respectively) consisted of the external validation cohort (24 females, 58.5%).

There was no significant difference in PFS between the two cohorts (median PFS: training cohort, 11.1 months; validation cohort, 13.1 months; *p* = 0.199). A hundred and forty-two of 172 (83%) patients developed disease progression during the follow-up period (109 patients in the training set, 33 patients in the external validation set). Fifteen of 172 (9%) patients didn’t progress (13 patients in the training set, 2 patients in the validation set) and 15 (9%) patients were lost to follow-up (9 patients in the training set, 6 patients in the validation set). The minimum first follow-up period was 16 days after therapy; while the maximum first follow-up time was 128 days (median: 35 days).

Furthermore, the two cohorts exhibited similar baseline clinical characteristics except for volume at baseline (*p =* 0.011). Details on the patient's characteristics as well as data available are provided in Table 1.

**Table 1:** Demographic and clinicopathologic characteristics

	Training (*n* = 131)	External (*n* = 41)	*p* value
Sex			0.960
Female	74 (56.5%)	24 (58.5%)	
Male	57 (43.5%)	17 (41.5%)	
Age			1.000
< 60	70 (53.4%)	22 (53.7%)	
≥ 60	61 (46.6%)	19 (46.3%)	
Smoking status			0.831
No	98 (74.8%)	32 (78.0%)	
Yes	33 (25.2%)	9 (22.0%)	
Ethnicity			0.246
Han Chinese	74 (56.5%)	28 (68.3%)	
Minority Chinese	57 (43.5%)	13 (31.7%)	
Tumor size (mm^3^)	29.7 (29.3)	20.2 (16.7)	0.011
T stage			0.089
I	5 (3.82%)	5 (12.2%)	
II	8 (6.11%)	4 (9.76%)	
III	16 (12.2%)	7 (17.1%)	
IV	102 (77.9%)	25 (61.0%)	
N stage			0.824
N0	16 (12.2%)	3 (7.32%)	
N1	34 (26.0%)	10 (24.4%)	
N2	48 (36.6%)	18 (43.9%)	
N3	33 (25.2%)	10 (24.4%)	
M stage			0.672
M0	5 (3.82%)	2 (4.88%)	
M1	126 (96.2%)	39 (95.1%)	
Clinical stage			0.462
I	0	0	
II	1	1	
III	1	1	
IV	129	39	
EGFR mutation status			0.729
Exon 19 deletion	62	20	
Exon 21 L858R	47	15	
Exon 18	4	0	
Exon 20	2	0	
EGFR+*	16	6	
TKI treatment			0.067
Afatinib	4 (3.05%)	1 (2.44%)	
Erlotinib	5 (3.82%)	1 (2.44%)	
Gefitinib	82 (62.6%)	17 (41.5%)	
Icotinib	33 (25.2%)	20 (48.8%)	
Osimertinib	7 (5.34%)	2 (4.88%)	
PFS	11.1 (8.22)	13.1 (8.43)	0.199

*Specific EGFR mutant type unknown

### Univariate and multivariate Cox regression

A univariate Cox regression analysis performed with all clinical factors indicated volume at baseline (HR: 1.006, CI: 1.000, 1.013, *p* = 0.045) as statistically significant to the prediction of disease progression (Table 2). Besides, other characteristics, such as sex, age, smoking status, TNM stage, etc., did not show significant prognostic impact. The clinical model constructed by baseline volume was the optimal model with the lowest AIC value (850.63).

**Table 2:** Univariate Cox regression analysis of clinical characteristics

	Hazard ratio	Confidence interval	*p* value
Sex			
Female	Ref		
Male	0.980	0.665, 1.445	0.920
Age			
< 60	Ref		
≥ 60	0.999	0.6853, 1.456	0.996
Smoking status			
No	Ref		
Yes	1.105	0.720, 1.696	0.648
Ethnicity			
Han Chinese	Ref		
Minority Chinese	0.810	0.555, 1.184	0.278
Tumor size (mm^3^)	1.006	1.000, 1.013	0.045
T stage			
I	Ref		
II	3.352	0.703, 15.98	0.129
III	2.518	0.571, 11.11	0.223
IV	1.696	0.416, 6.92	0.462
N stage			
N0	Ref		
N1	1.669	0.847, 3.292	0.139
N2	1.881	0.961, 3.683	0.065
N3	1.423	0.706, 2.869	0.324
M stage			
M0	Ref		
M1	0.453	0.142, 1.447	0.181
TKI treatment			
Afatinib	Ref		
Erlotinib	0.299	0.057, 1.575	0.154
Gefitinib	0.541	0.132, 2.223	0.394
Icotinib	0.540	0.127, 2.298	0.405
Osimertinib	0.182	0.025, 1.297	0.089

### Radiomics feature selection

In the training cohort, we extracted 6 sets of radiomics features from the intratumoral and peritumoral region (including peri_5mm_ ring and peri_10mm_ ring) at baseline and first follow-up CT scan, respectively. Each set comprised 1691 features that included 17 shape features, 324 first-order features, and 1350 texture features. The test-retest analysis refined the number of features to 6578 (each timepoint included 1162 intratumoral features, 1068 peri_5mm_ features and 1059 peri_10mm_ features). The baseline, follow-up, and delta radiomics features underwent the Boruta selection algorithm, and the ten most important features were combined for the final PFS-predicting signature using the random forest survival model. Among these features, 6 texture features, 3 shape-based, and one first-order feature were representatives. In terms of extraction location and time phase, 8 features were obtained from the peritumoral region and delta radiomics features accounted for 70% of the top 10 features.

### Model performance

**Fig. 3:**
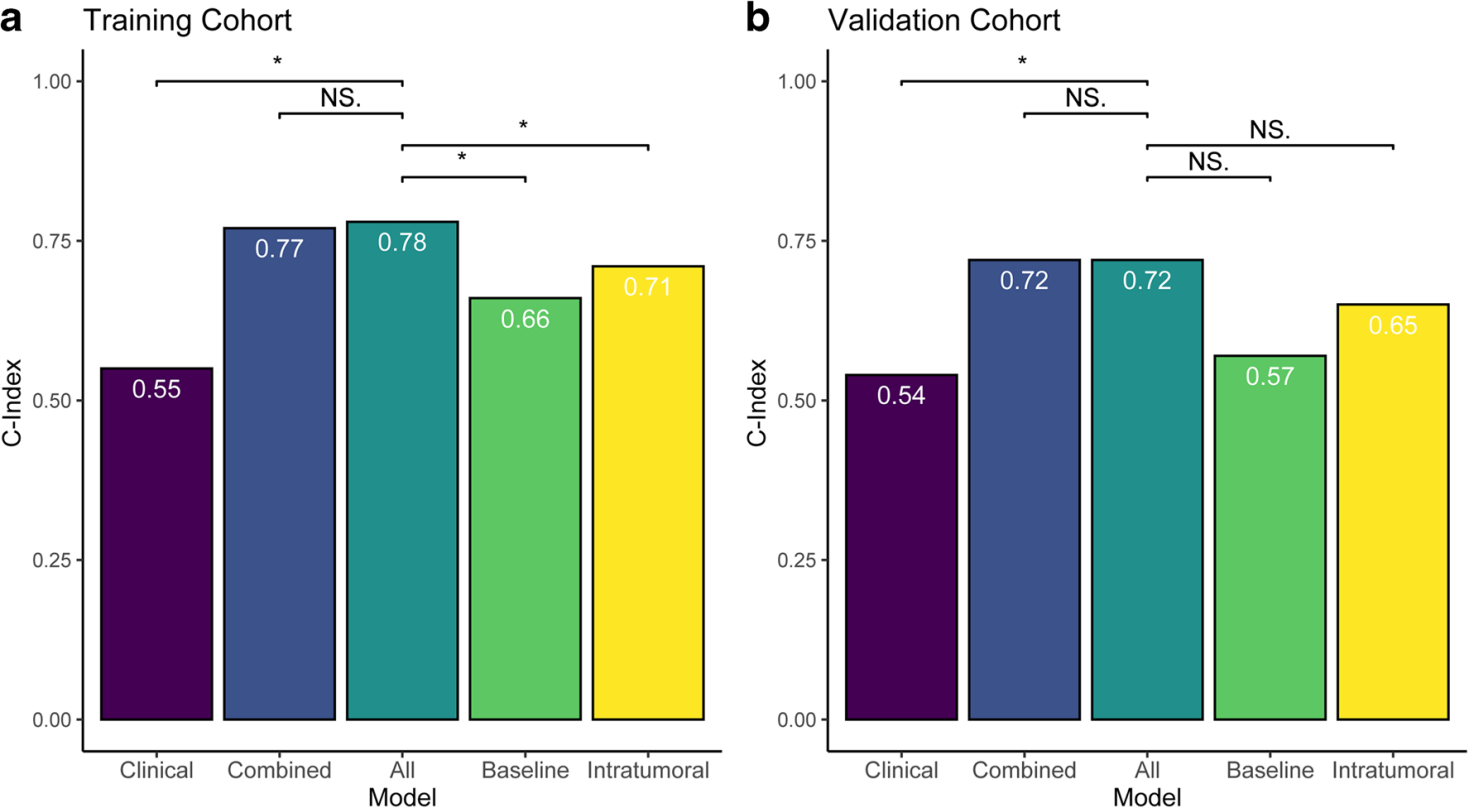
Model performance within training (**a**) and validation (**b**) cohorts. C-indices were compared among models. Asterisk (*) indicates statistical significance (*p* < 0.05) and NS denotes non-significance

The model_all_ for predicting PFS achieved the C-Index of 0.78 (95% CI: 0.72, 0.84) in the training cohort and 0.72 (95% CI: 0.60, 0.84) in the external validation cohort. The C-index yielded from the model_clinical_ was 0.55 (95% CI: 0.49, 0.62) in the training cohort and 0.54 (95% CI: 0.42, 0.66) in the validation cohort, which was both significantly lower than those of the model_all_ (*p* < 0.001). The combined clinico-radiomics model achieved the C-Index of 0.77 (95% CI: 0.71, 0.84) in the training cohort and 0.72 (95% CI: 0.60, 0.84) in the validation cohort. The composite model indicated no incremental benefit in progression prediction of both cohorts (*p* = 0.713, 0.756 respectively). The C-Index of the model_baseline_ (training cohort: 0.66, 95% CI:0.60, 0.73; validation cohort: 0.57, 95% CI:0.50, 0.69) and the C-Index of the model_intratumor_ (training cohort: 0.71, 95% CI:0.65, 0.78; validation cohort: 0.65, 95% CI:0.53, 0.77) were both numerically inferior to the comprehensive model. The comprehensive signature statistically outperformed the baseline and intratumoral signatures in the training cohorts (*p* < 0.001 and *p* = 0.006, respectively) but failed to reach statistical significance in the external validation cohort (*p* = 0.086 and *p* = 0.198, respectively) due to a limited sample size. The results of the model comparison were displayed in Fig. 3.

### Risk stratification and prognostic accuracy

**Fig. 4:**
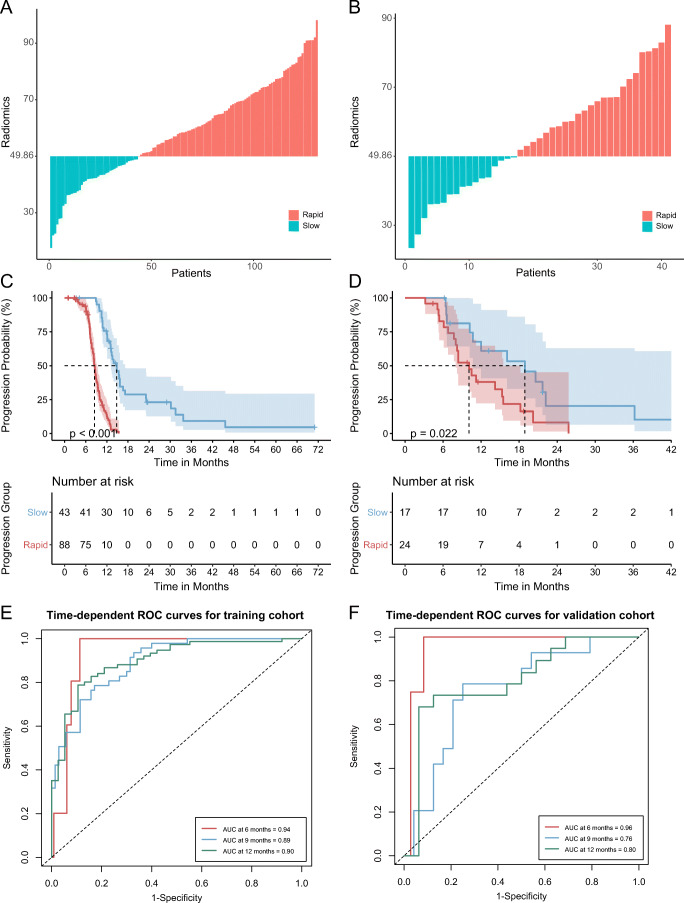
Risk stratification (**A**, **B**). The cutoff value of 49.86 was determined in training cohort by the log-rank statistics and used to classify the patients into rapid and slow progression subgroups. Kaplan–Meier survival (**C**, **D**) indicated significant difference in PFS between the two subgroups. Time-dependent ROC curves (**E**–**F**) displayed the predictive accuracy of progression at different time points. (**A**, **C**, **E**, training cohort; **B**, **D**, **F**, external validation cohort)

The log-rank statistics determined the optimal cutoff value of 49.86 (Figure S1) and divided the patients into rapid and slow progression subgroups (Fig. 4A, B). In both cohorts, the rapid progression group (median PFS: training cohort, 8.3 months; validation cohort, 9.1 months) had a shorter PFS than the slow progression group (median PFS: training cohort, 13.4 months; validation cohort, 13.2 months). There was a significant difference in PFS between the two groups based on Kaplan–Meier survival curves (Fig. 4C, D) in both cohorts.

The time-dependent ROC curves (Fig. 4E, F) demonstrated that the proposed radiomics signature (model_all_) was able to accurately and consistently predict the probability of disease progression at different time points. The AUCs at 6, 9, and 12 months ranged from 0.89 to 0.94 in the training cohort and 0.76 to 0.96 in the testing cohort.

## Discussion

Disease progression is a turn of events for LUAD patients undergoing EGFR-TKI therapy due to the inevitable presence of acquired resistance to TKIs. However, the detection of progression often lags behind its occurrence in clinical practice and timely detection remains challenging. In our study, we proposed a radiomics signature that accounted for the intra- and peri-tumoral characteristics along with their variation between serial follow-up examinations. As the result, the novel imaging signature allowed for early progression prediction and risk stratification in LUAD patients treated with EGFR-TKIs, potentially offering valuable reference information to determine disease progression.

Our study demonstrated a robust performance of the radiomics signature as evidenced by the promising C-index in both cohorts (training cohort: 0.78, validation cohort: 0.72). The result suggested that the CT-based radiomics signature could be used as a prognostic predictor for LUAD patients who received EGFR-TKI therapy. A multi-center study focusing on Stage IV EGFR-mutant NSCLC patients treated with EGFR-TKI therapy reported that the nomogram integrating pretherapy CT-based signature improved the prediction accuracy of PFS significantly [20]. A noteworthy difference from our work was the introduction of additional timepoint and our signature reflected the dynamic change of tumor heterogeneity. Tumor heterogeneity not only changes over time owing to subclonal diversities and genomic instability [36] but also alters between pre- and post-treatment under potent selective pressures arising from antineoplastic therapy, especially in targeted therapies [37, 38]. Fave et al discovered among 107 NSCLC patients during radiation therapy sessions that all radiomics features significantly changed from pretreatment CT imaging to subsequent weekly follow-ups [24]. Therefore, it could be insufficient to evaluate and predict individual progression status based on a single timepoint. Our study further confirmed that multi-timepoint radiomics signature outperformed the single-timepoint signature in PFS prediction in training cohort (C-index: 0.78 vs 0.66, *p* value < 0.001). The result highlighted the incremental value of dynamic tumor characteristics for this specific patient population.

Among the ten features constituting our radiomics signature, seven delta-radiomics features contributed crucial information associated with patient-specific outcome prediction. Previous studies presented controversy over the calculation and application of these delta-radiomics features. Nardone et al discovered delta-texture features were more robust than texture features through a phantom study [39]. A retrospective study of 107 NSCLC patients under radiotherapy revealed that pretreatment imaging features were not prognostic, whereas delta-radiomics features had a statistically significant impact on estimation for local recurrence [24]. Contrarily in another smaller study consisting of 48 EGFR-mutant LUAD patients who received EGFR-TKIs, follow-up features were more predictive for PFS than the percentage change [21]. We suspected that different duration between therapy initiation and first follow-up imaging among patients could create bias. Therefore, we adjusted such factor by calculating the unit-time (per day) percentage change between the two timepoints, and our data indeed proved the prognostic value of these dynamic characteristics.

The interface between primary tumor and lung parenchyma offered clues to how tumor cells interact with recruited immune and stromal cell of the surrounding normal tissue [40–42]. Under such interplay, tumor cells acquired migratory phenotype and the capability of invading adjacent tissue [43–45]; therefore, capturing such phenotypic expression in the peritumoral regions could be beneficial. Our findings suggested the model integrating intra- and peritumoral features produced superior PFS predictive power over the intratumoral-alone model in training cohort (C-index: 0.78 vs 0.71, *p* value = 0.006). The prognostic benefit of peritumoral features was echoed by Dou et al in their study that peritumoral radiomics features were shown to have better prognostic performance compared to tumor radiomic signature for distant metastasis in patients with locally advanced NSCLC [26].

Baseline clinical characteristics exhibited weak association with progression status in the studied cohorts, as evidenced by the univariate analysis. The clinical model alone could not accurately predict PFS (C-index in training cohort: 0.55, validation cohort: 0.54); moreover, adding clinical characteristics to radiomics signature did not reveal any improvement. It suggested primary tumor volume at baseline was of limited value in prognostication of disease progression for LUAD patients undergoing EGFR-TKIs. Our result was consistent with several previous studies [20, 21] that baseline volume had a lower discriminative power in PFS estimation for EGFR-mutant LUAD patients receiving EGFR-TKIs compared with radiomics features.

The application of EGFR TKIs could result in different disease progression patterns and variable PFS. Kim et al [21] discovered that the PFS ranged from 0.3 to 29.4 months for the 48 enrolled lung adenocarcinoma patients treated with EGFR-TKI. Meanwhile, in another study, Song et al [20] found that the median PFS was 5.6 and 10.7 months for EGFR-TKI-treated NSCLC patients with rapid and slow progression, respectively. RECIST 1.1 assessment depends on the size change of the target lesion or appearance of new lesions, and disease progression can only be confirmed at the CT images on which progression has already occurred. The radiomics signature proposed in our study also accounted for lesion heterogeneity in the intratumoral and peritumoral space, along with their dynamic change throughout the course of therapy. As the result, we could make an early prognosis at the first follow-up imaging. We determined a cutoff value of the radiomics signature to stratify patients into rapid progression group (R-group) and slow progression groups (S-group). The stark survival contrast between groups potentially suggested personalized follow-up strategies; for instance, patients predictive of rapid progression may benefit from more positive and rigorous follow-up. The time-dependent ROC analysis further confirmed a favorable prognosis throughout the disease course, indicating accurate detection of progression and better re-biopsy timing selection.

There were some limitations in our study due to the inherent nature of its retrospective study. Firstly, the sample size was relatively small, particularly in the external validation cohort. Geographical variations also potentially introduced biases between the two studied cohorts. Our results need further verification on more large-scale cohorts. Secondly, CT imaging acquisition was derived from multiple institutes, different CT scanners, and protocols. The diagnostic efficacy of radiomics features can be influenced by the variability of CT acquisition and reconstruction parameters [46–51]. To alleviate the negative effect of scanner variability, we normalized slice thickness through 1 × 1 × 1 mm resampling aiming to achieve more standardized feature extraction. Thirdly, unlike several previous studies, we analyzed non-contrast CT images rather than contrast-enhanced CT scans. Despite the added hemodynamic information, contrast agent could contaminate the biological heterogeneity within tumors depicted by radiomics features [46, 52]. Since NSCLC patients undergoing EGFR-TKI therapy require long-term follow-up, non-contrast CT may be more appropriate due to its high cost-effectiveness, strong reproducibility and controllable adverse effects and complications.

In conclusion, the proposed integrated radiomics signature was capable of capturing the dynamic information of intra- and peritumoral heterogeneity for LUAD patients who received EGFR-TKI therapy. This comprehensive imaging signature demonstrated considerably high potential in the prediction of disease progression and risk stratification. Once validated in larger cohorts, it is conducive to guide clinical decision-making such as personalized follow-up strategies.

## Supplementary Information

ESM 1 (DOCX 126 kb)

## Abbreviations

3D: Three-dimensional
AIC: Akaike information criterion
AUC: Areas under the curve
CI: Confidence interval
C-index: Concordance index
EGFR: Epidermal growth factor receptor
HIS: Hospital Information System
ICC: Interclass correlation coefficient
IQR: Interquartile ranges
LUAD: Lung adenocarcinoma
NCCN: National comprehensive cancer network
NSCLC: Non-small cell lung cancer
PACS: Picture Archiving and Communication Systems
PFS: Progression-free survival
R-group: Rapid progression group
ROC: Receiver operating characteristic
S-group: Slow progression group
TKI: Tyrosine kinase inhibitor
VOI: Volume of interest

## Declarations

### Guarantor

The scientific guarantor of this publication is Liling Long, E-mail: cjr.longliling@vip.163.com.

### Methodology

• retrospective

• prognostic study

• multicenter study
