# Supplementary material for: Prognostic analysis and risk stratification of lung adenocarcinoma undergoing EGFR-TKI therapy with time-serial CT-based radiomics signature
Source: Eur Radiol. 2022 Sep 27;33(2):825–35. doi: 10.1007/s00330-022-09123-5 (PMC9889474; doi:10.1007/s00330-022-09123-5)
Supplement: Supplementary file 1 — (DOCX 126 kb) [file 330_2022_9123_MOESM1_ESM.docx]

Supplementary material

Table S1 CT acquisition and reconstruction parameters

|  | Manufacturer | | KV | mA | Slice thickness(mm) | Rotation time(sec) | SFOV(mm) | Pixel matrix | Convolution  Kernel |
| --- | --- | --- | --- | --- | --- | --- | --- | --- | --- |
| *BLINDED* | SIEMENS | SOMATOM Definition Flash | 100/120/140 | 60-777 | 1, 1.5, 2, 8 | 0.5-1 | 500 | 512×512 | B20f/B22f/B30f/B31f  /B41f/B50f  I26f/I30f |
|  |  | SOMATOM Perspective | 110/130 | 60-328 | 1, 1.5, 2 | 0.5-1 | 500 | 512×512 | I41s/B41s |
|  |  | SENSATION 16 | 120 | 266/272 | 2 | 0.5-1 | 500 | 512×512 | B30f |
|  |  | SOMATOM Force | 100/110 | 109-245 | 1 | 0.5-1 | 500 | 512×512 | Br40d |
|  | GE | Revolution CT | 100/120/140 | 69-399 | 1.25, 2.5, 5 | 0.5-1 | 500 | 512×512 | STANDARD |
|  |  | LightSpeed VCT | 100/120 | 80-360 | 0.625, 1.25, 2.5, 3.75, 5 | 0.5-1 | 500 | 512×512 | STANDARD |
| *BLINDED* | SIEMENS | SOMATOM Definition Flash | 100/120 | 94-347 | 1, 2 | 0.5-1 | 500 | 512×512 | B20f/B22f/B31f  I26f/I31f/I50f |
|  | Philips | iCT 256 | 120 | 100-276 | 1, 2 | 0.5-1 | 500 | 512×512 | B |
| *BLINDED* | GE | Revolution CT | 100/120 | 105-627 | 5 | 0.5-1 | 500 | 512×512 | STANDARD |
|  |  | Optima CT660 | 120 | 55-499 | 1.25, 5 | 0.5-1 | 500 | 512×512 | STANDARD |
|  |  | LightSpeed VCT | 120 | 203-516 | 1.25 | 0.5-1 | 500 | 512×512 | STANDARD |

Table S2 Radiomics feature composition

|  | **Original** | **LoG filtering** | **Wavelet filtering** | **Non-linear intensity transforms** | **Total** |
| --- | --- | --- | --- | --- | --- |
|  |  | 0.5, 1.5, 2.5,  3.5, 4.5 mm | LLL, LLH, LHL, LHH, HLL, HLH, HHL, HHH | square, square root,  logarithm, exponential |  |
| **Shape** | 17 |  |  |  | 17 |
| **First-order** | 18 | 18 × 5 | 18 × 8 | 18 × 4 | 324 |
| **Texture** | 75 | 75 × 5 | 75 × 8 | 75 × 4 | 1350 |
| **Total** | 110 | 465 | 744 | 372 | 1691 |


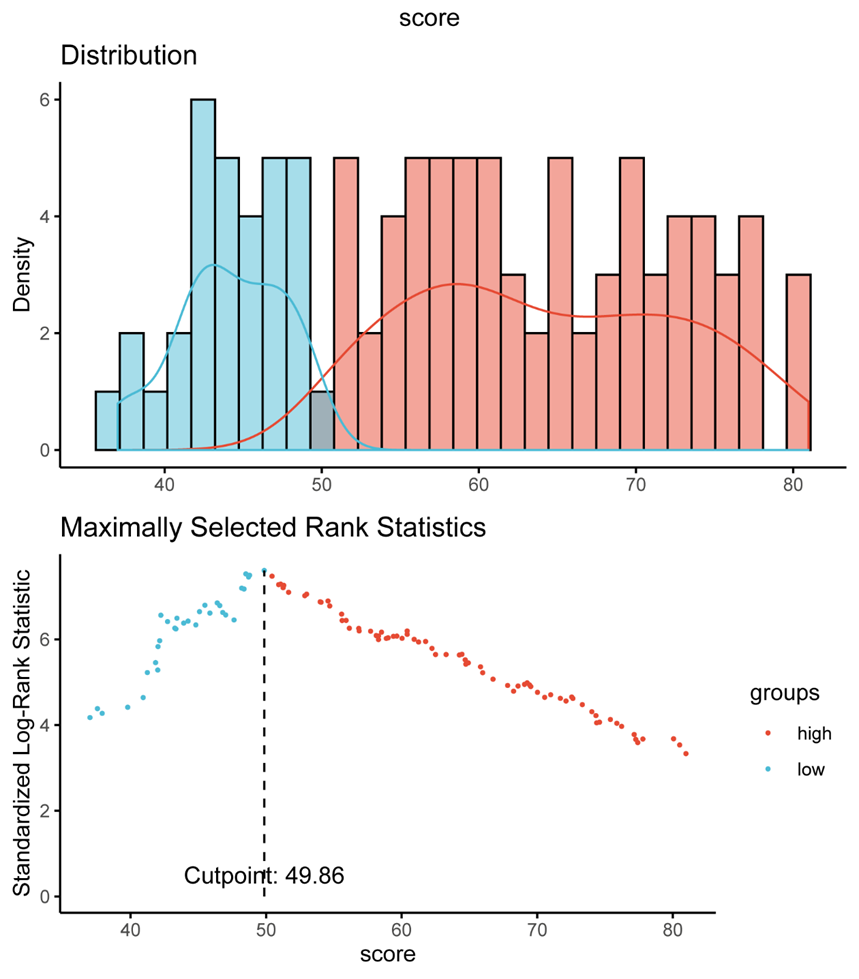


Figure S1. Cutoff determination for the radiomics signature score based on the training cohort. The score distribution of the 131 patients in the training cohort is displayed in histogram (upper row), and maximal standardized log-rank statistics corresponds to the optimal cutoff score of 49.86 (lower row).
